# Accumulation of Cadmium in and Its Effect on the Midgut Gland of Terrestrial Snail *Helix pomatia* L. from Urban Areas in Poland

**DOI:** 10.1007/s00128-014-1346-y

**Published:** 2014-08-13

**Authors:** Tadeusz Włostowski, Paweł Kozłowski, Barbara Łaszkiewicz-Tiszczenko, Ewa Oleńska, Olgierd Aleksandrowicz

**Affiliations:** Institute of Biology, University of Białystok, Świerkowa 20B, 15-950 Białystok, Poland

**Keywords:** Cadmium, *Helix pomatia*, Midgut gland, Metallothionein, Lipid peroxidation, Lipofuscin

## Abstract

The objectives of this study were (1) to determine cadmium (Cd) accumulation in the midgut gland of a land snail *Helix pomatia * L. inhabiting residential areas of the 14 largest cities in Poland, and (2) to examine whether the accumulated Cd exerted any toxic effects. The average accumulation of Cd in the midgut gland of snails, weighing 16–18 g, ranged from 7.00 to 87.3 µg/g dry weight (0.06–0.77 µmol/g) and differed significantly among animals from the various urban areas. This difference in Cd accumulation was not related to city population, but was associated with the topsoil Cd (R^2^ = 0.868, *p* < 0.0001). The tissue Cd was not found to produce toxicity (histopathology, programmed cell death, lipofuscin formation or lipid peroxidation), probably due to the induction of sufficiently high quantities of metallothionein and glutathione, well-known protective molecules.

Cadmium (Cd) is an environmental pollutant ranked seventh in the priority list of hazardous substances (ATSDR 2007). In humans and animals, Cd can damage various organs and tissues, including kidney, liver, lung, pancreas, testis and bone (Wittman and Hu 2002; Satarug et al. 2003; Zalups and Ahmed 2003; Järup and Akesson 2009). The metal is also classified as a category I human carcinogen (Weisberg et al. 2003). Unfortunately, its amount in the environment still increases mainly due to human activities such as the combustion of coal and oil to produce heat and electricity, non-ferrous metal manufacturing, the production of plastics, alloys, iron and cement, and waste incineration (Wittman and Hu 2002; Pacyna et al. 2007). These activities are generally located in urban areas which, therefore, become more contaminated with Cd than other areas (Sawicka-Kapusta et al. 2003). This is also evidenced by a substantial increase of Cd concentration in the urban soils (Ajmone-Marsan and Biasoli 2010; Luo et al. 2012). However, the impact of soil contamination on human health and soil organisms, especially in residential and recreational areas, is not well studied and remains to be elucidated.

Land snails, including *Helix pomatia * L., have been shown to exhibit a high capacity to accumulate Cd in the midgut gland (hepatopancreas) (Dallinger and Wieser 1984; Rabitsch 1996; Menta and Parisi 2001; Dallinger et al. 2004), which makes them useful tools for the assessment of the metal pollution in soil (Dallinger 1994). The high capacity of Cd accumulation is related directly to efficient induction of a specific Cd-binding metallothionein (MT) isoform which is responsible for retention and detoxification of the metal in the midgut gland of *H. pomatia* and other species (Dallinger et al. 1997, 2004; Chabicovsky et al. 2004; Hödl et al. 2010). It has been demonstrated further that pathological alterations such as increased programmed cell death and cell proliferation, the formation of lipofuscin granules, and the disruption of mitochondrial membranes in this organ can occur at Cd concentrations above a threshold of 0.8 µmol/g dry weight, when all binding sites on the MT isoform are saturated with Cd and the resulting non-MT-bound Cd ions can exert their toxic effects (Hödl et al. 2010). One mechanism by which these ions can produce pathology in vertebrates and invertebrates is thought to be through generation of reactive oxygen species and oxidation of membrane lipids and proteins (Roesijadi et al. 1997; Liu et al. 2009; Amachree et al. 2013). So far, however, little is known about the accumulation and toxicity of Cd in the midgut gland of *H. pomatia* snails ranging in an urban area.

Therefore, the purposes of this study were (1) to determine Cd accumulation in the midgut gland of *H. pomatia* inhabiting residential areas of the 14 largest cities in Poland, and determine whether this accumulation was related to city population size and soil Cd, and (2) to examine whether the accumulated Cd affected tissue structure, programmed cell death, the formation of lipofuscin granules and lipid peroxidation. In addition, the concentrations of MT and glutathione (GSH) that are linked to a protective effect against Cd toxicity (Chan and Cherian 1992; Nzengue et al. 2008) were determined.

## Materials and Methods

**Fig. 1:**
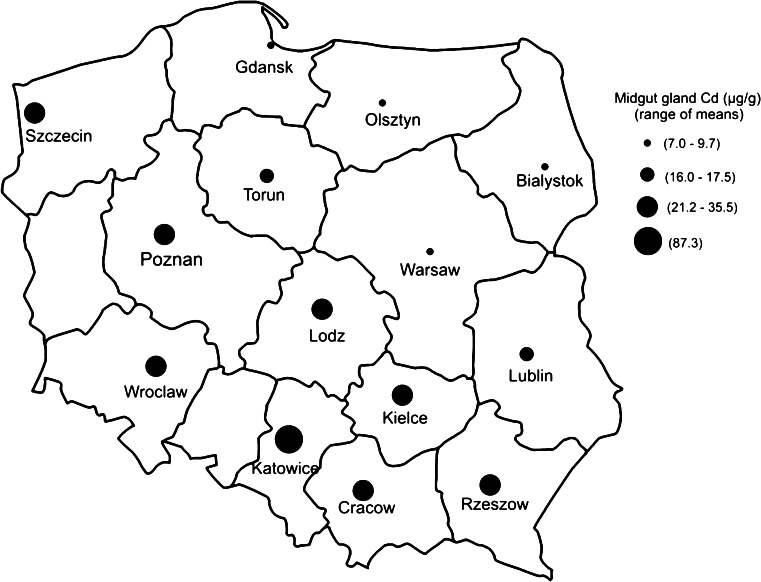
Map of Poland showing location of the 14 sampling areas. Sizes of *black circles* indicate relative cadmium accumulation in the midgut gland of *Helix pomatia* snails under study

Ten individuals of *H. pomatia*, weighing 16–18 g, were collected from each of the 14 cities in Poland (Fig. 1) in the period of 15 June–15 July 2011. The snails were sampled from three different sites located in a residential area (gardens, parks) of each city. In addition, from each site the topsoil (0–5 cm) as composite sample (500 g) was taken for determination of Cd and pH. In the laboratory, the soil samples were dried at 60°C, and then crushed and passed through a 1-mm sieve. Soil pH was determined in soil suspensions (1:2.5 soil to water ratio) using a pH meter. The snails were decapitated, and the midgut gland was removed and divided into three portions. One portion (about 200 mg wet weight) was dried at 60°C for 48 h and used for determination of Cd and water (to calculate a dry weight conversion factor). Another one was fixed in 4% formaldehyde for histological examination. The third portion was frozen and kept at −80°C until analysis of MT, GSH and lipid peroxidation. After thawing, a portion (about 250 mg) of this organ was transferred to 1.0 mL chilled 0.25 M sucrose and homogenized with a Teflon pestle in a glass homogenizer. An aliquot (0.2 mL) was taken for determination of lipid peroxidation. The remaining homogenate was centrifuged at 20,000×*g* for 20 min at 4°C, and the resulting supernatant was removed for MT and GSH assays. The data were expressed on a dry weight basis, using dry weight conversion factors.

Cadmium content in the midgut gland of *H. pomatia* snails was determined as described in Włostowski et al. (2010). Briefly, a portion of the organ (60–70 mg dry weight) was placed in a glass tube and 2.0 mL of redistilled nitric acid (70%) (Sigma-Aldrich) was added. After 20 h of sample digestion at room temperature, 72% perchloric acid (0.5 mL) was added and the mixture was heated at 100°C for 3 h. Finally, the temperature was raised to 150–180°C and digestion continued for another 3 h. Deionized water was added to the residue after digestion to a volume of 3.0 mL (first solution). A portion of the first solution (200 µL) was evaporated to dryness in a quartz crucible at 130°C, and the residue was redissolved in an appropriate amount of deionized water (second solution). Cd analyses of these solutions were carried out by electrothermal atomic absorption spectrometry (AAS) using a Thermo Sollar M6 instrument with Zeeman correction (ThermoFisher Scientific, Waltham, MA, USA). A standard solution (Sigma-Aldrich, Poznan, Poland) was used to prepare the standard curve. Quality assurance procedures included the analysis of reagent blanks and standard reference material (Bovine liver 1577c—National Institute of Standards and Technology, Gaithersburg, MD). The limit of detection in the acid digest (3 × standard deviation of 10 reagent blanks plus the mean) was 2.00 µg/L. For a 60 mg of tissue the detection limit was 0.10 µg/kg dry weight. The precision expressed as relative standard deviation (RSD) of 10 measurements of the same sample was 5% and the recovery of Cd was 91%–96%.

In the case of soil Cd, 0.5 g of the soil was extracted with 2.0 mL of concentrated nitric acid in a closed polypropylene tube for 5 days, and then heated at 70°C for 24 h (Dallinger et al. 2004). During extraction, the tubes were repeatedly shaken by hand. After this treatment the soil suspensions were centrifuged at 30,000×*g* for 30 min, and the supernatants were transferred to a quartz crucible and evaporated to dryness. The residue was redissolved in an appropriate amount of deionized water and analyzed for Cd by electrothermal AAS. The analysis of each soil sample was done in triplicate.

MT in the midgut gland was determined by a Cd-saturation method and expressed in µg Cd bound to MT/g dry weight (Włostowski et al. 2010). The total GSH (reduced + oxidized) was measured according to the method of Tietze (1969) by using a glutathione assay kit (NWLSS, Vancouver, WA, USA). Lipid peroxidation was assessed by measuring malondialdehyde (MDA) formation, using the thiobarbituric acid (TBA) assay (Ohkawa et al. 1979).

**Fig. 2:**
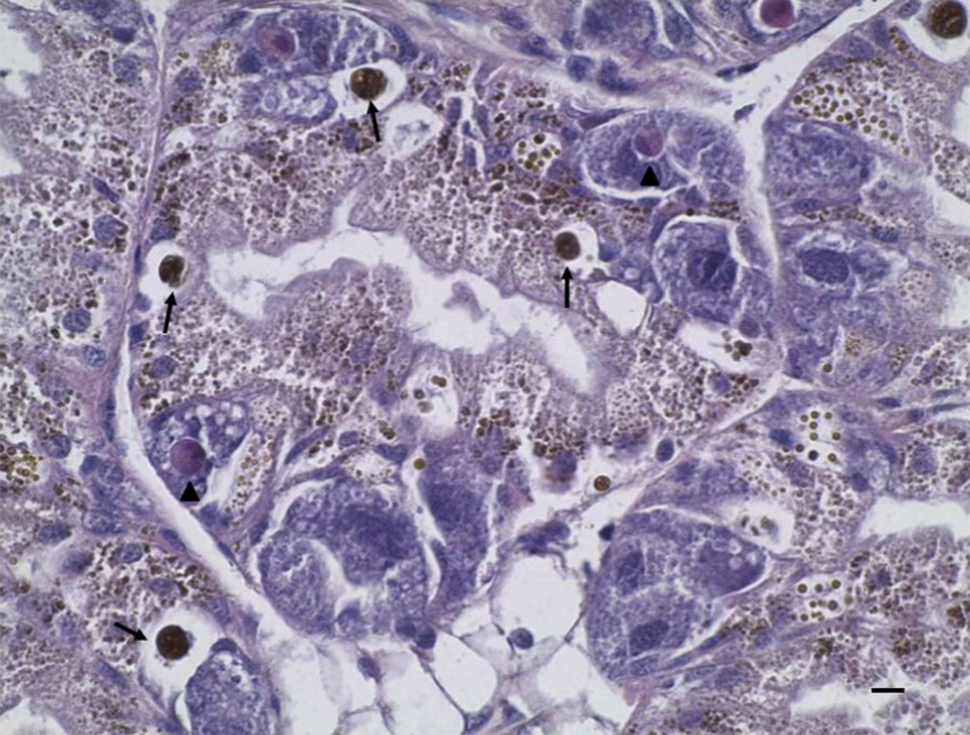
Representative photomicrograph of a midgut gland section from the *Helix pomatia* snail under study. Note the residual bodies (lipofuscin granules) within large vacuoles of excretory cells (*arrows*) and the condensed body (programmed cell death) situated in a basophilic cell (*arrow head*). *Scale bar* 20 µm

The fixed portions of the midgut gland were dehydrated in ethanol and xylene, embedded in paraffin, cut into 5 µm sections (Leica microtome), and stained with hematoxylin and eosin for microscopic examination. The number of cells with lipofuscin granules (Fig. 2) were determined in ten random microscopic fields for each snail, using a 40 × objective, and the results were expressed as the mean number of cells with lipofuscin granules per microscopic field. Also condensed bodies (programmed cell death) (Fig. 2) (Chabicovsky et al. 2004) were counted in the same sections.

Data were expressed as mean ± SD. They were analyzed by one-way analysis of variance (ANOVA) followed by the Duncan’s multiple range test (when required, the variables were normalized using log_10_ transformation). Differences at *p* < 0.05 were considered statistically significant. The simple regression analysis was used to examine the relationship between Cd accumulation in the midgut gland and the number of inhabitants and soil Cd, as well as between the midgut gland Cd and the MT, GSH, lipid peroxidation and the number of cells with lipofuscin granules. All the statistical analyses were performed using IBM SPSS Statistics 21 program (IBM Corporation, Somers, NY, USA).

## Results and Discussion

**Fig. 3:**
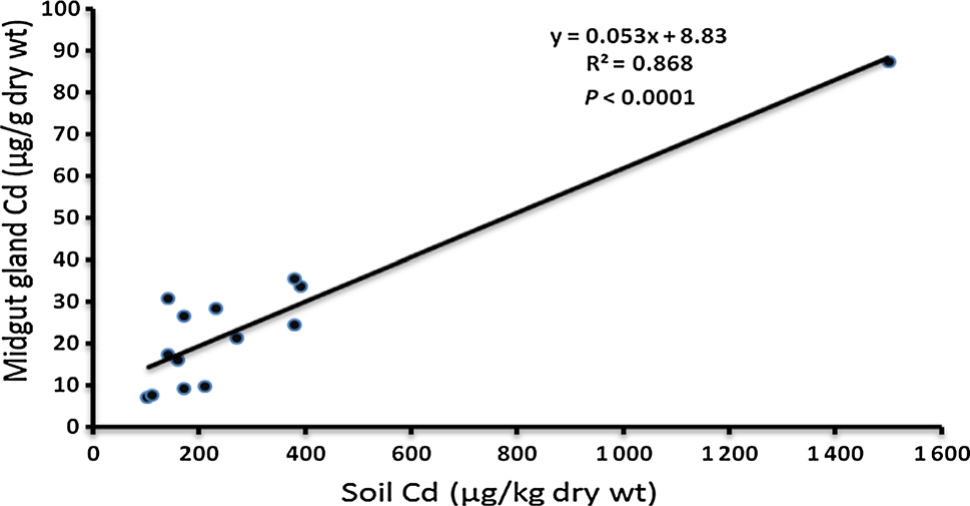
The relationship of the topsoil Cd to the midgut gland Cd in *Helix pomatia* snails

The concentrations of Cd in the midgut gland of *H. pomatia* snails and in the soil from a residential area of 14 cities are presented in Table 1. The levels of Cd in the midgut gland differed significantly (*p* < 0.0001) among snails living in the different cities. The lowest levels of the metal (means below 10 µg/g dry wt) were found in 4 cities (Bialystok, Olsztyn, Gdansk and Warsaw), the highest (87 µg/g dry wt) was noted in Katowice, and medium concentrations (16–35 µg/g dry wt) were observed in animals from the remaining 9 cities (Fig. 1; Table 1). This difference in Cd accumulation was not related to the weight (age) of animals, which was similar (16–18 g) in the snails from all cities. Also, the size of a city (number of inhabitants) (Table 1) did not affect the accumulation of Cd in the midgut gland (R^2^ = 0.029, *p* > 0.5). The simple regression analysis revealed, however, that the accumulation of Cd in this organ correlated significantly with the topsoil Cd (R^2^ = 0.868, *p* < 0.0001) (Fig. 3), but the soil Cd was not related to the size of a city (R^2^ = 0.01). Although soil pH has been demonstrated to affect Cd accumulation in snails (Pauget et al. 2012), this factor could only have a negligible effect in the *H. pomatia* snails because the soil pH (6.95–7.35) was almost identical in all cities.

**Table 1:** The levels of soil cadmium (Cd), and Cd level, metallothionein, glutathione, lipid peroxidation (TBARS) and residual bodies in the midgut gland of *Helix pomatia* snails from the largest cities in Poland

City	Number of inhabitants (thousand)*	Soil Cd (µg/kg)	Midgut gland Cd (µg/g)	Metallothionein (µg Cd/g)	Glutathione (µmol/g)	TBARS (nmol/g)	Residual bodies**
Olsztyn	176	100 ± 30^a^	7.00 ± 3.19^a^	8.80 ± 3.40^a^	0.17 ± 0,17^a^	257 ± 43^a^	3.2 ± 0.6^a^
Rzeszow	178	230 ± 50^ab^	28.4 ± 13.2^b^	30.0 ± 13.0^b^	0.34 ± 0.30^a^	232 ± 38^a^	3.3 ± 0.7^a^
Kielce	204	390 ± 60^b^	33.6 ± 13.0^b^	37.2 ± 14.0^b^	0.31 ± 0.20^a^	153 ± 48^b^	6.0 ± 0.8^b^
Torun	205	140 ± 30^a^	17.5 ± 3.40^c^	19,4 ± 5.60^c^	0.31 ± 0.27^a^	288 ± 46^a^	3.4 ± 0.5^a^
Bialystok	295	110 ± 40^a^	7.75 ± 3.38^a^	10.8 ± 3.00^a^	0.27 ± 0.17^a^	158 ± 27^b^	3.8 ± 0.5^a^
Katowice	307	1,500 ± 450^c^	87.3 ± 17.4^d^	99.2 ± 16.0^d^	0.68 ± 00.7^b^	215 ± 26^a^	6.0 ± 2.0^b^
Lublin	348	160 ± 40^ab^	16.0 ± 3.00^c^	17.6 ± 2.40^c^	0.41 ± 0.31^ab^	231 ± 41^a^	6.2 ± 0.7^b^
Szczecin	406	380 ± 50^b^	24.4 ± 10.8^bc^	25.3 ± 11.0^bc^	0.24 ± 0.17^a^	232 ± 36^a^	4.1 ± 1.5^ab^
Gdansk	457	170 ± 50^ab^	9.11 ± 5.80^a^	10.1 ± 6.20^ac^	0.20 ± 0.17^a^	253 ± 58^a^	5.1 ± 1.6^ab^
Poznan	552	140 ± 30^a^	30.7 ± 9.40^b^	32.2 ± 9.70^b^	0.20 ± 0.16^a^	211 ± 71^ab^	6.4 ± 1.8^b^
Wroclaw	633	270 ± 90^ab^	21.2 ± 12.4^abc^	22.6 ± 13.0^abc^	0.37 ± 0.31^ab^	254 ± 48^a^	2.5 ± 1.3^a^
Lodz	737	170 ± 30^ab^	26.5 ± 13.7^bc^	27.4 ± 13.6^bc^	0.41 ± 0.37^ab^	165 ± 21^b^	5.4 ± 1.5^ab^
Cracow	756	380 ± 80^b^	35.5 ± 7.20^b^	38.6 ± 10.0^b^	0.92 ± 0.41^b^	218 ± 13^a^	4.2 ± 1.0^a^
Warsaw	1,720	210 ± 80^ab^	9.67 ± 3.41^a^	10.3 ± 4.50^ac^	0.41 ± 0.15^a^	163 ± 23^b^	5.0 ± 1.0^ab^

Data are presented as mean ± SD for n = 10 (gland) and n = 3 (soil). Means in the same column marked with a different superscript letter are significantly different (*p* < 0.05) (ANOVA and Duncan’s multiple range test)

* After GUS (2011)

** Number of cells with lipofuscin granules/microscopic field

The midgut gland was also analyzed for MT and GSH contents (Table 1). The Cd-binding capacity of MT in this organ followed a pattern similar to that of Cd concentration, and the capacity exceeded the total concentration of this metal in the snails from all urban areas (Tables 1, 2). Furthermore, the concentration of GSH in the midgut gland correlated positively with Cd concentration (Table 2), suggesting that this compound could also be induced by the metal.

**Table 2:** The correlation between cadmium, and metallothionein (MT), glutathione (GSH), lipid peroxidation (TBARS) and residual bodies (RB) in the midgut gland of *Helix pomatia* snails (n = 14, means from Table 1 were compared)

Comparison	Equation	R^2^	*p*
MT versus Cd	MT = − 0.88 + 1.10 × Cd	0.98	0.0000
GSH versus Cd	GSH = 0.23 + 0.006 × Cd	0.46	0.0296
TBARS versus Cd	TBARS = 221 − 0.18 × Cd	0.008	0.7660
RB versus Cd	RB = 3.9 + 0.02 × Cd	0.14	0.1877

In this work, the toxicity of Cd was evaluated by assessing histopathology, programmed cell death (condensed bodies) and lipofuscin formation (residual bodies) (Fig. 2), as well as lipid peroxidation (Table 1). No histopathological change (necrosis) was observed, and evidence of programmed cell death was very rarely observed in the midgut gland of all snails from the 14 cities. These data were not suitable for statistical analysis. Residual bodies were observed exclusively in the excretory cells (Fig. 2), and their means differed significantly (*p* < 0.05) amongst the cities (Table 1). However, there was no significant relationship of the tissue Cd to the lipofuscin formation (Table 2). Likewise, lipid peroxidation (an index of oxidative stress) in the midgut gland differed significantly (*p* < 0.05) among snails from the various cities (Table 1), but did not correlate significantly with tissue Cd (Table 2).

The present work demonstrated that Cd accumulation in the midgut gland of *H. pomatia* from an urban area was strongly related to topsoil Cd. Thus, these data confirm the notion that *H. pomatia* snails are suitable animals for the assessment of Cd contamination in soil (Dallinger 1994). The current data indicate further that the accumulation of Cd in the midgut gland is not associated with the population of a city, which suggests that the inhabitants do not affect the soil and tissue concentrations of Cd. Indeed, the levels of Cd in the soil and midgut gland of *H. pomatia* from the largest city (Warsaw) were similar to those from the smaller ones (Bialystok, Gdansk, Olsztyn), and significantly lower than those from other cities (Table 1). Thus, it may be concluded that the differences in Cd concentration among the urban areas are linked to other factors; for instance, a different natural concentration of Cd in the bedrock and/or industrial emission of Cd to the atmosphere may play an important role (Luo et al. 2012). The first possibility may be confirmed by the fact that the concentrations of Cd in the soil and midgut gland of *H. pomatia* from Kielce (204,000 inhabitants) are 2–3-fold higher than those from Warsaw (1,720,000 inhabitants) although the emission of Cd to the atmosphere from the territory of the two districts is similar (about 100 g/km^2^/year) (Hławiczka 2008). On the other hand, the highest concentrations of Cd observed in Katowice (Fig. 1; Table 1) are probably due to long-term emission of Cd to the atmosphere, which is the highest in Poland and now amounts to 600 g/km^2^/year (Hławiczka 2008). This pollution is probably caused by heavy industry situated in this area which includes, among others, coal-based power station and heating units, hard coal mines, an iron factory and a non-ferrous metal smelter (Kennedy et al. 1999). However, irrespective of Cd origin (natural or anthropogenic) the lowest environmental levels of this metal are characteristic of Olsztyn, Bialystok, Gdansk and Warsaw (north-eastern part of Poland), slightly higher for Lublin and Torun, 3–5-fold higher for Cracow, Kielce, Lodz, Poznan, Rzeszow, Szczecin and Wroclaw, and highest for Katowice (Fig. 1). In this regard it is worth noting that the renal Cd levels in non-smoking inhabitants from Katowice, Cracow and Lodz have been shown to be significantly higher than those found in people from Bialystok (Piotrowski et al. 1996). These data suggest that environmental Cd in the urban areas affects the tissue Cd not only in *H. pomatia* snails but also in humans. In the case of humans, both house dust and the consumption of home-grown crops are considered to be important routes of exposure to Cd (Järup and Akesson 2009; Ajmone-Marsan and Biasoli 2010).

The average accumulation of Cd in the midgut gland of *H. pomatia* from the 14 cities ranged from 7.00 to 87.3 µg/g dry weight (0.06–0.77 µmol/g) (Table 1). It has been recently demonstrated that pathological changes in the midgut gland of *H. pomatia* such as the increasing formation of lipofuscin granules occurs only at Cd concentrations above a threshold of 0.8 µmol/g dry weight (90 µg/g) (Hödl et al. 2010). Thus, it can be concluded that observations of pathological effects in the midgut gland of free-ranging snails in these urban areas would be unlikely. Indeed, there was no histopathology and only single cells undergoing programmed cell death were observed; in addition, no correlation was found between the midgut gland Cd and the number of cells with lipofuscin granules (Table 2), suggesting the lack of toxic effects of the metal accumulated in this organ. No toxic effects of Cd in the midgut gland could have resulted from the Cd induction of appropriate amounts of MT (Tables 1, 2). It is well known that the Cd-MT isoform of *H. pomatia* is involved in Cd detoxification (Dallinger et al. 1997; Hödl et al. 2010) and its Cd-binding capacity in the midgut gland of snails from all cities exceeded the total concentration of Cd, which indicated that the non-MT-bound Cd (if any) was too low to exert toxic effects. In addition, GSH that is also known to provide a protection against Cd toxicity (Chan and Cherian 1992; Nzengue et al. 2008) increased in relation to Cd concentration in the midgut gland, thereby preventing Cd-induced oxidative stress; no correlation between lipid peroxidation and Cd concentration in the gland suggested that this may have been the case. It cannot be ruled out, however, that the induction of lipid peroxidation by Cd in the snails may occur at much higher concentrations of this metal than those required for GSH or MT induction. Nevertheless, no Cd toxicity observed in the midgut gland of *H. pomatia* may be linked to sufficiently high concentrations of the two protective molecules.

In conclusion, the results of this study showed that the accumulation of Cd in the midgut gland of *H. pomatia* snails from urban areas was directly related to topsoil Cd concentration, which was not related to city population size. Relatively high levels of Cd in the midgut gland of snails from some urban areas appeared not to produce toxic responses, most probably due to the induction of appropriate quantities of MT and GSH.
